# Matrix Stiffness Regulates TGFβ1‐Induced αSMA Expression via a G9a‐LATS‐YAP Signaling Cascade

**DOI:** 10.1096/fba.2025-00117

**Published:** 2025-07-14

**Authors:** Chinmay S. Sankhe, Jessica L. Sacco, Victoria L. Crunkleton, Malcom Díaz García, Matthew J. Bierowski, David Vidotto Rezende Soares, Jacob A. Karnick, Rachel L. Cecco, Arefeh Abbasi, Joy Kirigo, Thomas K. Wood, Esther W. Gomez

**Affiliations:** ^1^ Department of Chemical Engineering Pennsylvania State University University Park Pennsylvania USA; ^2^ Department of Materials Science and Engineering North Carolina State University Raleigh North Carolina USA; ^3^ Department of Mechanical Engineering University of Puerto Rico Mayagüez Puerto Rico USA; ^4^ Penn State College of Medicine Pennsylvania State University Hershey Pennsylvania USA; ^5^ Department of Biomedical Engineering Pennsylvania State University University Park Pennsylvania USA

**Keywords:** epigenomics, epithelial cells, extracellular matrix, histone methyltransferases, hydrogels, mammary gland

## Abstract

Extracellular matrix stiffness is enhanced in cancer and fibrosis; however, there is limited knowledge on how matrix mechanics modulate expression and signaling of the methyltransferase G9a. Here, we show that matrix stiffness and transforming growth factor (TGF)‐β1 signaling together regulate G9a expression and the levels of the histone mark H3K9me2. Suppressing the activity and expression of G9a attenuates TGFβ1‐induced alpha smooth muscle actin (αSMA) and N‐cadherin expression and cell morphology changes in mammary epithelial cells cultured on stiff substrata. Knockdown of G9a increases the expression of large tumor suppressor kinase 2 (LATS2) and decreases the nuclear localization of yes associated protein (YAP). Furthermore, inhibition of LATS promotes an increase in YAP nuclear localization and αSMA expression, while inhibition of YAP attenuates αSMA expression. Overall, our findings indicate that a G9a‐LATS‐YAP signaling cascade regulates mammary epithelial cell response to matrix stiffness and TGFβ1.

## Graphical abstract

A G9a‐LATS‐YAP signaling cascade regulates epithelial‐mesenchymal plasticity in mammary epithelial cells in response to matrix stiffness and TGFβ1.

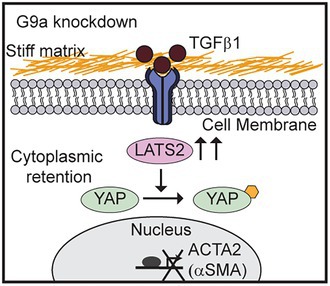

## 1 Introduction

Transforming growth factor (TGF)‐β1 regulates development and homeostasis, and dysregulation of TGFβ signaling can contribute to pathologies including fibrosis and cancer. During fibrotic and tumor progression, the extracellular matrix experiences changes in composition, organization, and mechanics [1]. Indeed, atomic force microscopy has revealed an increase in tissue stiffness in idiopathic pulmonary fibrosis [2], breast cancer [3, 4], and other types of cancer in comparison to normal tissue [5].

TGFβ1 is a potent inducer of epithelial‐mesenchymal transition (EMT), which is characterized by a loss of the expression of epithelial markers, including E‐cadherin, and a gain in the expression of mesenchymal markers, including N‐cadherin, vimentin, and alpha smooth muscle actin (αSMA). These gene expression changes are accompanied by changes in cell morphology and cytoskeletal organization, which together enable cells to acquire a more motile phenotype. Within human breast tumors, markers of EMT colocalize with regions of the tumor that exhibit high stiffness [6]. Furthermore, previous studies show that mammary [7, 8, 9, 10], lung [11], kidney [9], and skin [12] epithelial cells cultured on stiff hydrogels exhibit gene expression changes that are consistent with EMT in response to TGFβ1. While these studies have revealed important roles for myocardin related transcription factors, phosphoinositide 3‐kinase (PI3K)/Akt, Smads, and the mechanosensitive channel transient receptor potential vanilloid 4 in mediating this response, mechanistically, how matrix stiffness works in concert with TGFβ1 to impact epigenetic remodeling and gene expression has not been fully elucidated.

Histone modifications regulate chromatin packaging and thereby can impact activation and inactivation of transcription. The methyltransferase G9a is primarily responsible for catalyzing dimethylation of histone 3 at lysine 9 (H3K9me2) which represses transcription [13, 14]. G9a mediates repression of anti‐fibrotic genes *COX‐2* and *CXCL10* [15, 16] and maintains fibroblast activation by silencing PPARγ coactivator 1α (PGC1α) thereby promoting lung fibrosis [17]. In the context of cancer, elevated G9a levels are associated with poor prognosis, and G9a contributes to silencing of tumor suppressor genes [18, 19, 20]. G9a also contributes to repression of epithelial markers such as Ep‐CAM and E‐cadherin, thereby promoting EMT and cancer cell invasion into the surrounding matrix [21, 22, 23]. Furthermore, inhibition of G9a activity decreases radiation‐induced expression of mesenchymal markers vimentin and N‐cadherin in lung epithelial cells [24]. Despite the importance of G9a in fibrosis and cancer, the impact of matrix stiffness on G9a expression and signaling in response to TGFβ1 is not known.

TGFβ signaling integrates with Hippo signaling pathway components yes‐associated protein 1 (YAP) and transcriptional coactivator with PDZ‐binding motif (TAZ) to mediate fibrosis and cancer [25, 26, 27]. YAP subcellular localization is regulated by large tumor suppressor kinases 1/2 (LATS1/2), which can phosphorylate YAP, thereby promoting cytoplasmic retention [26], as well as by actomyosin tension, which can promote its nuclear localization [28, 29]. In the nucleus, YAP interacts with transcription factors such as TEAD to regulate a variety of genes including αSMA [30, 31]. Decreased expression of LATS1/2 promotes cell migration in normal mammary gland epithelial cells, suggesting a role for this pathway in EMT [32]. Interestingly, in human cholangiocarcinoma cells, G9a suppresses LATS2 expression levels [33]. This previous study suggests that epigenetic signaling via G9a can operate together with intracellular signaling of LATS‐YAP to drive disease progression. However, whether G9a regulates Hippo signaling pathway components in response to matrix stiffness and TGFβ1 stimulation is yet to be investigated.

Here, we utilized hydrogels with stiffnesses that mimic the mechanical properties of mammary tissue to examine the role of G9a in mediating mammary epithelial cell response to TGFβ1. We find that an increase in matrix stiffness and TGFβ1 signaling together modulate G9a mRNA and protein expression and the levels of the H3K9me2 histone mark in normal mammary gland epithelial cells. Additionally, we show that G9a activity and expression regulate TGFβ1‐induced αSMA and N‐cadherin expression in response to increasing matrix stiffness. G9a modulates LATS2 and YAP in cells cultured on a stiff matrix, and this correlates with regulation of TGFβ1‐induced αSMA expression by matrix stiffness. Our findings suggest that targeting G9a and downstream signaling molecules could be used as an approach to downregulate the expression of mesenchymal markers in pathological contexts of fibrosis and cancer.

## 2 Materials and Methods

### 2.1 Synthesis of Polyacrylamide Hydrogels

Polyacrylamide (PA) hydrogels were prepared following an adapted protocol [7, 34]. Briefly, acrylamide (Sigma Aldrich) and bis‐acrylamide (Sigma Aldrich) were mixed in water, and the solution was degassed. Ammonium persulfate (10% w/v, Sigma Aldrich) and N, N, N′, N′‐tetramethylethylenediamine (TEMED; 0.05% v/v, Sigma Aldrich) solutions were added to initiate the polymerization reaction. The solution was then pipetted onto glass slides which were treated with 0.1 N sodium hydroxide, 2% v/v aminopropyltrimethoxysilane (APTMS; Sigma Aldrich), and glutaraldehyde (Sigma Aldrich). The solution was allowed to polymerize for 45–60 min.

To facilitate cell attachment to the PA hydrogels, the surfaces of the hydrogels were activated with 0.5 mM N‐sulfosuccinimidyl‐6‐(4′‐azido‐2′ nitrophenylamino) hexanoate (Sulfo‐SANPAH; Thermo Scientific) diluted in 50 mM HEPES buffer at pH 8.5. The PA hydrogels were exposed to ultraviolet light in a CL‐1000 Ultraviolet Crosslinker (UVP) for 10 min. The hydrogel surfaces were rinsed with 50 mM HEPES buffer (pH 8.5), and the activation process was repeated. Following activation, the PA hydrogels were rinsed extensively with 50 mM HEPES buffer (pH 8.5) and treated with 10 μg/mL human fibronectin (BD Biosciences) overnight at 4°C. Hydrogels were then washed three times with sterile 1× phosphate buffer saline (PBS) to remove excess fibronectin prior to plating cells.

### 2.2 Rheology

The mechanical properties of the hydrogels were characterized using an RFS‐3 rheometer (Rheometric Scientific Inc., Model number ARES‐3). Time sweeps were performed at a frequency of 1 rad/s and 2% strain, and frequency sweeps were performed at 2% strain. The storage modulus, *G*′, is reported at a frequency of 1 rad/s, which corresponds to the reported frequency at which cell mechanosensing occurs [35]. The Young's modulus (E) was calculated from the storage modulus using $E=2G\prime \left(1+\vartheta \right)$ where ϑ is the Poisson ratio. Poisson's ratio for polyacrylamide has been reported to range between 0.3 and 0.48 [36, 37, 38, 39].

### 2.3 Cell Culture and Inhibitor Reagents

Normal murine mammary gland (NMuMG) epithelial cells were obtained from American Type Culture Collection (ATCC Cat# CRL‐1636, RRID: CVCL_0075) and were maintained in Dulbecco's Modified Eagle Medium (DMEM; Corning) with 10% (v/v) fetal bovine serum (FBS; Atlanta Biologicals), 50 μg/mL gentamicin (Gibco), and 10 μg/mL insulin (Sigma Aldrich). NMuMG cells were authenticated by Genetica Cell Line Testing, a LabCorp brand, and tested negative for mycoplasma. Madin Darby Canine Kidney (MDCK) epithelial cells were obtained from American Type Culture Collection (ATCC Cat# CCL‐34, RRID: CVCL_0422) and maintained in Eagle's Minimum Essential Medium (MEM; Corning) supplemented with 10% (v/v) FBS and 50 μg/mL gentamicin. Cells were cultured in complete growth medium within a humidified incubator set at 37°C and 5% CO_2_. Cells were seeded onto fibronectin‐coated PA hydrogels at a density of ~40,000 cells/cm^2^ and EMT was induced by treating the cells with 10 ng/mL of recombinant human TGFβ1 (R&D Systems) for 48 h. Carrier solution (1 mg/mL bovine serum albumin in 4 mM HCl) was used as a control. For inhibitor studies, cells were treated with UNC0642 (10 nM; Selleck Chemicals), verteporfin (4 μM; Sigma Aldrich), or TRULI (15 μM, Sigma Aldrich) diluted in dimethyl sulfoxide (DMSO) for 1 h prior to treatment with TGFβ1.

MCF10A human mammary epithelial cells (ATCC Cat# CRL‐10317, RRID: CVCL_0598) were grown at 37°C and 5% CO_2_ in a humidified incubator in DMEM/F12 media (Fisher) supplemented with 5% horse serum (Fisher), 20 ng/mL hEGF (Thermo), 0.5 μg/mL hydrocortisone (Sigma), 100 ng/mL Cholera toxin (Sigma), 10 μg/mL human insulin (Sigma), and 10 U/mL Pen/Strep (VWR). When cells were seeded onto the hydrogels, media was switched to low serum media (2% horse serum and 5 ng/mL hEGF). MCF10A cells were treated with 10 ng/mL recombinant human TGFβ1 (R&D Systems) or a vehicle control for 72 h.

### 2.4 siRNA Transfections

siRNA targeting G9a (predesigned siRNA Assays siG9a#1: 90322 and siG9a#2: 90229; AM16708) and negative control siRNA (AM4390843) were obtained from Thermo Fisher Scientific. Cells were plated in 6‐well plates at a density of 300,000 cells/well and transfected with 25 pmol of siRNA constructs using Lipofectamine RNAiMAX transfection reagent (Life Technologies) and Opti‐MEM medium following the manufacturer's recommended protocol. A siRNA transfection efficiency of ~85% was confirmed using BLOCK‐iT Alexa Fluor Red Fluorescent Control (Invitrogen). Knockdown of G9a following transfection with siRNA was confirmed by western blotting, and siG9a#2 demonstrated strong knockdown of G9a. Cells transfected with non‐targeting control siRNA, siG9a#1, or siG9a#2 were seeded onto fibronectin‐coated PA hydrogels 24 h post transfection. Cells were cultured on hydrogels for approximately 24 h and then treated with TGFβ1 or control vehicle solution for another 48 h prior to further experimental analysis.

### 2.5 Immunofluorescence Staining

For staining of αSMA, cells cultured on PA hydrogels were rinsed with 1× PBS and fixed in 1:1 methanol: acetone at −20°C for 10 min. For staining of E‐cadherin, G9a, YAP, LATS2, and H3K9 methylation markers, cells were rinsed in 1× PBS and fixed in 4% paraformaldehyde at room temperature for 15 min. After fixation, samples were treated with 0.5% v/v IGEPAL (Sigma) and permeabilized with 0.1% v/v Triton X‐100 each for 10 min. Samples were then blocked with 5% goat serum (Sigma) or 5% bovine serum albumin (BSA; Sigma) in 1× PBS for 1 h and incubated with primary antibodies overnight at 4°C. Samples were rinsed in 1× PBS thrice to remove unbound primary antibody before incubating with secondary antibodies at room temperature for 1 h. Samples were again rinsed with 1× PBS thrice to remove unbound secondary antibody, and the nuclei of cells were counterstained with Hoechst 33342 nuclear dye (Life Technologies) before mounting the samples on microscope slides using Fluoromount‐G (Invitrogen). Antibodies that were used for immunofluorescence staining are as follows: H3K9me2 (1:200; Abcam Cat# ab1220, RRID: AB_449854), E‐cadherin (1:200; Cell Signaling Technology Cat# 3195, RRID: AB_2291471), αSMA (1:200; Sigma‐Aldrich Cat# A5228, RRID: AB_262054), G9a (1:500; D5R4R, Cell Signaling Technology Cat# 68851, RRID: AB_2799755), YAP (1:100; D8H1X, Cell Signaling Technology Cat# 14074, RRID: AB_2650491), LATS2 (1:100; Sigma‐Aldrich Cat# SAB4501146, RRID: AB_10745837), and Alexa Fluor secondary antibodies (1:500; Alexa Fluor 594 goat anti‐rabbit (Invitrogen A‐11037) and Alexa Fluor 488 goat anti‐mouse (Invitrogen A‐11029)).

### 2.6 Microscopy and Image Analysis

Stained samples were imaged using a 20× objective on a Nikon Eclipse Ti‐E inverted fluorescence microscope equipped with a Photometrics CoolSNAP HQ^2^ CCD camera. Cells transfected with BLOCK‐iT Alexa Fluor oligos were imaged on a Leica DMi8 inverted microscope with a K8 camera. Imaging conditions and settings were kept constant for all sample treatments. Integrated fluorescence intensities were measured using ImageJ (RRID: SCR_003070) or CellProfiler 4.2.8 Image Analysis Software (RRID: SCR_007358). For analysis of the levels of histone markers and G9a protein, at least 50 nuclei were analyzed per treatment condition, and the total integrated intensity within individual cell nuclei was measured. For analysis of the levels of LATS2, the total integrated intensity within individual cells was measured. Intensities were normalized to the control treatment condition. For computing the percentage of cells expressing αSMA, the number of cells staining positive for αSMA was counted and divided by the total number of cells examined. For YAP nuclear localization measurements, the total integrated intensity of YAP was computed inside the nucleus and within the entire cell by outlining the boundary of the nucleus and the cell in ImageJ. Cytoplasmic YAP intensity was computed by subtracting nuclear integrated intensity from the whole cell integrated intensity. YAP nuclear intensity 1.5‐fold greater than cytoplasmic intensity within cells was classified as nuclear localized YAP.

### 2.7 Western Blotting

Histones were extracted from cells cultured on 45‐mm diameter circular PA hydrogels using an acid extraction protocol as described previously [40]. Histone protein concentration was quantified with a Bradford assay using Coomassie Plus Assay Reagent (Thermo Scientific). For whole cell protein extractions, cells were lysed using ice‐cold RIPA buffer containing Halt protease and phosphatase inhibitors (Thermo Scientific) and the protein concentration was quantified using a Pierce BCA Protein Assay Kit (Thermo Scientific). Protein samples were denatured using NuPAGE LDS sample buffer (Invitrogen) and NuPAGE sample reducing agent (Invitrogen) at 70°C for 10 min. Equal amounts of protein were separated using gel electrophoresis on a NuPAGE 4%–12% bis‐tris gel (Invitrogen) at 120 V, using NuPAGE (2‐(N‐morpholino) ethanesulfonic acid) MES SDS running buffer (Invitrogen) in a XCell SureLock Cell powered by a Bio‐Rad PowerPac HC. The proteins on the gel were transferred to a nitrocellulose or PVDF membrane using a XCell II Blot Module at 30 V for 1 h. A solution of 5% w/v non‐fat dry milk or 5% w/v BSA in 1× Tris‐buffered saline plus 0.1% Tween (TBST) was used to block the membrane. The membranes were probed with primary antibodies overnight at 4°C. Membranes were then washed thrice with 1× TBST buffer and incubated with IRDye secondary antibodies at room temperature for 1 h, and then re‐washed thrice with 1× TBST. Western blots were imaged using a Licor Odyssey imaging system. Blot images were analyzed using ImageJ software, and densitometric analysis was performed. Antibody dilutions used for western blotting are as follows: H3K9me2 (1:1000; Abcam Cat# ab1220, RRID: AB_449854), E‐cadherin (1:1000; 24E10, Cell Signaling Technology Cat# 3195, RRID: AB_2291471), αSMA (1:1000; Sigma‐Aldrich Cat# A5228, RRID: AB_262054), N‐cadherin (1:1000; 13A9, Cell Signaling Technology Cat# 14215, RRID: AB_2798427), G9a (1:1000; D5R4R, Cell Signaling Technology Cat# 68851, RRID: AB_2799755), YAP (1:1000, 1A12, Cell Signaling Technology Cat# 12395, RRID: AB_2797897), pYAP‐S127 (1:1000, D9W2I, Cell Signaling Technology Cat# 13008, RRID: AB_2650553), GAPDH (1:1000, D16H11, Cell Signaling Technology Cat# 5174, RRID: AB_10622025), αtubulin (1:1000, DM1A, Cell Signaling Technology Cat# 3873, RRID: AB_1904178), and IRDye secondary antibodies (1:15000; IRDye 680rd, LI‐COR Biosciences Cat# 925–68,071, RRID: AB_2721181 and 1:15000; IRDye 800 CW, LI‐COR Biosciences Cat# 926–32,210, RRID: AB_621842).

### 2.8 Quantitative Real‐Time (qRT)‐PCR

Total RNA was extracted using an RNeasy Plus Mini Kit (Qiagen) according to the manufacturer's protocol, and RNA quality was assessed using a nanodrop spectrophotometer. qRT‐PCR was performed using an iTaq Universal SYBR Green One‐Step Kit (Bio‐Rad) according to the manufacturer's protocol. Forward and reverse primers used are listed in Table S1. GAPDH was used as a normalization control. For all experiments, mRNA levels were normalized to the levels of GAPDH mRNA and to the levels of the soft hydrogel vehicle control samples.

### 2.9 Statistical Analysis

Data are reported as mean ± standard error of the mean (sem), and all experiments were repeated three times, unless otherwise noted. A two‐tailed student's *t*‐test or analysis of variance (ANOVA) followed by Tukey's post hoc test was performed using Minitab (RRID: SCR_014483) or GraphPad Prism (RRID: SCR_002798) for sample comparison. For data that was not normally distributed, including single cell measurements of cell spread area and aspect ratio, the non‐parametric Kruskal‐Wallis test with Dunn's post hoc analysis was performed using GraphPad Prism version 9.5.1 (RRID: SCR_002798). Differences were considered significant for *p* values less than 0.05 and are indicated as *p < 0.05, **p < 0.01, ***p < 0.001, and ****p < 0.0001.

## 3 Results

### 3.1 Matrix Stiffness and TGFβ1 Regulate G9a Expression and H3K9 Dimethylation Levels

Atomic force microscopy has revealed that mammary tumors exhibit a heterogeneous stiffness distribution with a soft peak centered around 570 Pa and a broader stiff peak centered around 5750 Pa [41]. To model mammary tumor tissue, we synthesized polyacrylamide hydrogels mimicking the stiffness of the soft and stiff peaks of the tumor stiffness distribution. Rheology revealed storage moduli (*G*′) of 260 ± 30 Pa and 2200 ± 70 Pa for the soft and stiff polyacrylamide hydrogels (Figure S1, Table S2). Assuming a Poisson ratio of 0.45 [39], the Young's moduli of these hydrogels are 750 ± 60 Pa and 6400 ± 170 Pa, respectively. Fibronectin, which is found in higher levels in the stroma of breast tumors than in normal mammary tissue [42, 43, 44] and stimulates EMT [45, 46], was used to functionalize the surfaces of the hydrogels to enable cell attachment.

**FIGURE 1:**
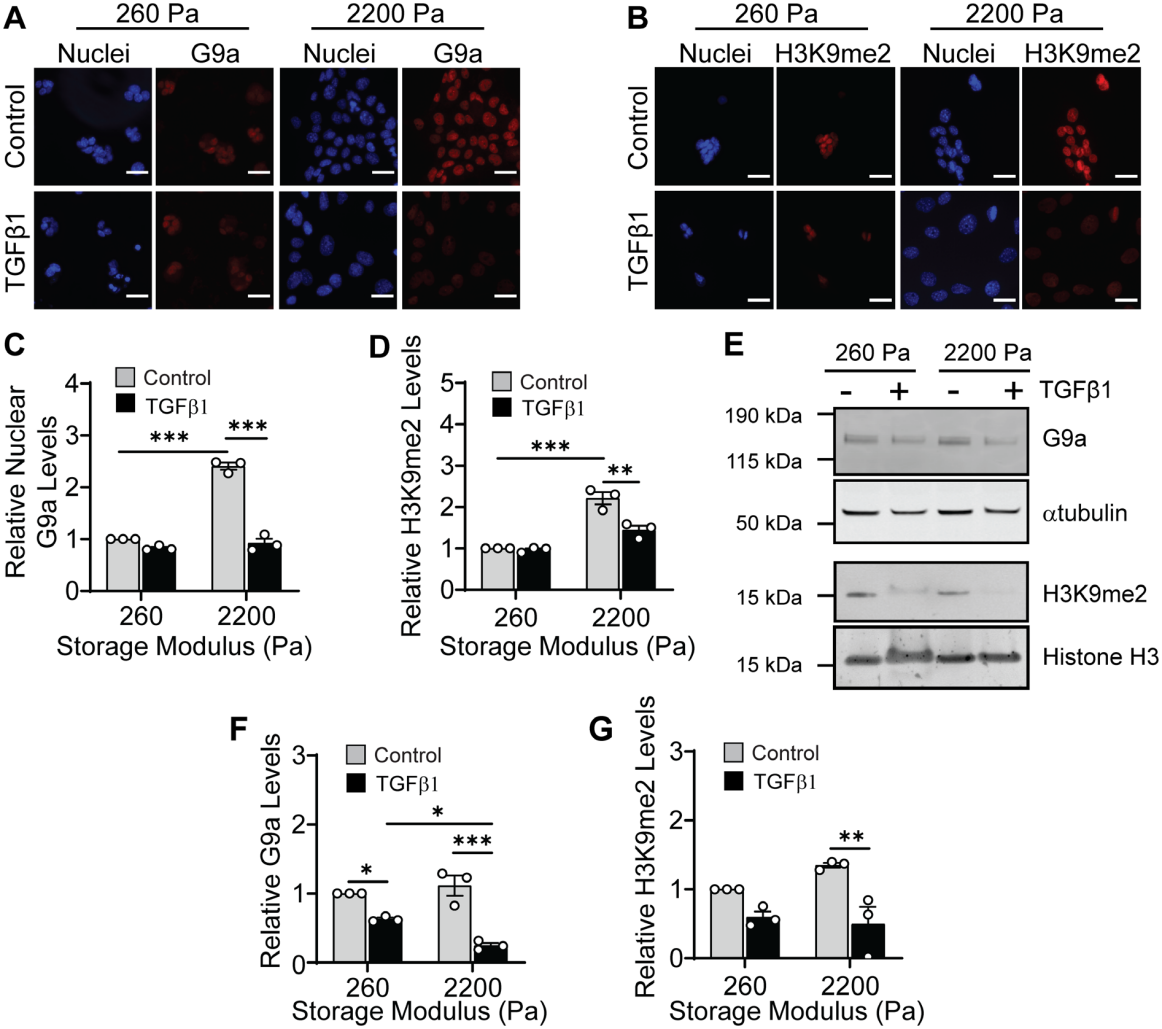
Matrix stiffness regulates G9a and histone 3 lysine 9 dimethylation in response to TGFβ1. Immunofluorescence staining for (A) G9a and (B) H3K9me2 in NMuMG cells cultured on hydrogels and treated with or without TGFβ1. Scale bars: 25 μm. Quantification of the (C) relative nuclear G9a and (D) relative H3K9me2 levels from immunofluorescence images shown in panels (A) and (B). Data are normalized with respect to the soft hydrogel control sample. (E) Western blot for G9a using whole cell protein extracts and H3K9me2 using histone extracts from NMuMG cells cultured on hydrogels with and without TGFβ1 treatment. Relative quantification via densitometric analysis for (F) G9a and (G) H3K9me2 from blots shown in panel (E). Data are normalized with respect to the soft hydrogel control sample. All data represent mean ± sem for *n* = 3 independent experiments; *p < 0.05, **p < 0.01, ***p < 0.001.

NMuMG cells were cultured on the fibronectin‐coated hydrogels and treated with TGFβ1 to induce EMT. G9a, a methyltransferase that contributes to epigenetic repression of anti‐fibrotic genes [15, 16] and tumor suppressor genes [18, 19, 20], mediates aspects of TGFβ‐induced EMT [23]. Examination of G9a localization using immunofluorescence staining revealed that G9a primarily localizes to the cell nucleus (Figure 1A). Quantification of G9a integrated intensity levels within individual cell nuclei shows a decrease in the levels of G9a following TGFβ1 treatment in cells cultured on stiff (*G*′ = 2200 Pa) hydrogels as compared to the vehicle control‐treated cells (Figure 1C). Western blotting of whole cell protein extracts revealed that TGFβ1 treatment decreases G9a levels in cells cultured on hydrogels with storage moduli of 260 Pa and 2200 Pa in comparison to control vehicle treated cells (Figure 1E,F). Additionally, the levels of G9a are significantly decreased in cells treated with TGFβ1 when cultured on 2200 Pa hydrogels in comparison to cells cultured on 260 Pa hydrogels. Moreover, qRT‐PCR revealed that TGFβ1 induces a decrease in G9a mRNA levels in cells cultured on both 260 Pa and 2200 Pa hydrogels in comparison to treatment with control vehicle (Figure S2A). Together, these observations suggest that G9a levels are regulated by a combination of matrix stiffness and TGFβ1 signaling.

G9a primarily catalyzes dimethylation of histone 3 at lysine 9 (H3K9me2) [47, 48, 49, 50]. To confirm the activity of G9a in cells cultured on hydrogels following TGFβ1 treatment, we examined H3K9me2 levels. Immunofluorescence staining for H3K9me2 revealed a decrease in H3K9me2 levels in cells cultured on 2200 Pa hydrogels when treated with TGFβ1 in comparison to treatment with control vehicle (Figure 1B,D). Western blotting revealed that, similar to the trend in the G9a levels, TGFβ1 treatment decreases the bulk levels of H3K9me2 in cells cultured on 2200 Pa hydrogels compared to the control vehicle treatment (Figure 1E,G). We also find that the bulk levels of H3K9me2 are reduced in TGFβ1‐treated cells cultured on 260 Pa hydrogels in comparison to the control vehicle treated cells. Similar to the trend in G9a levels, however, the decrease in levels is not found to be statistically significant.

To confirm that regulation of G9a and H3K9me2 levels in response to matrix stiffness and TGFβ1 treatment is not cell type dependent, we also performed western blotting for G9a in MCF10A human mammary epithelial cells and immunofluorescence staining for H3K9me2 in Madin Darby canine kidney (MDCK) epithelial cells. As in the case of NMuMG cells, we observe a decrease in G9a levels in MCF10A cells following treatment with TGFβ1 (Figure S2B,C). Furthermore, a decrease in H3K9me2 levels is observed following TGFβ1 treatment in MDCK cells cultured on 2200 Pa hydrogels (Figure S2D,E). Overall, these findings suggest that matrix stiffness and TGFβ1 signaling regulate the levels of H3K9me2, which is likely controlled by G9a methyltransferase activity.

### 3.2 Inhibition of G9a Activity Attenuates TGFβ1‐Induced EMT in Response to Matrix Stiffness

Previous results have shown that matrix stiffness regulates TGFβ1‐induced EMT in mammary epithelial cells [7, 8, 9, 10]. To elucidate the role of G9a in regulating TGFβ1‐induced EMT in response to matrix stiffness, we suppressed the activity of G9a using the inhibitor UNC0642 [51]. During EMT, cells exhibit marked shifts in phenotype, including changes in cell morphology, cytoskeletal organization, and gene expression [52]. To examine the impact of inhibition of G9a on cell morphology, we quantified cell spread area and cell aspect ratio. When cells are cultured on stiff (*G*′ = 2200 Pa) hydrogels, TGFβ1 treatment promotes an increase in cell spread area and elongation in comparison to cells treated with the vehicle control (Figure S3A,B). While treatment with UNC0642 does not affect TGFβ1‐induced cell spreading in cells cultured on stiff hydrogels, it does promote a decrease in cell aspect ratio (Figure S3A,B). For cells cultured on soft (*G*′ = 260 Pa) hydrogels, UNC0642 treatment does not affect cell morphology.

**FIGURE 2:**
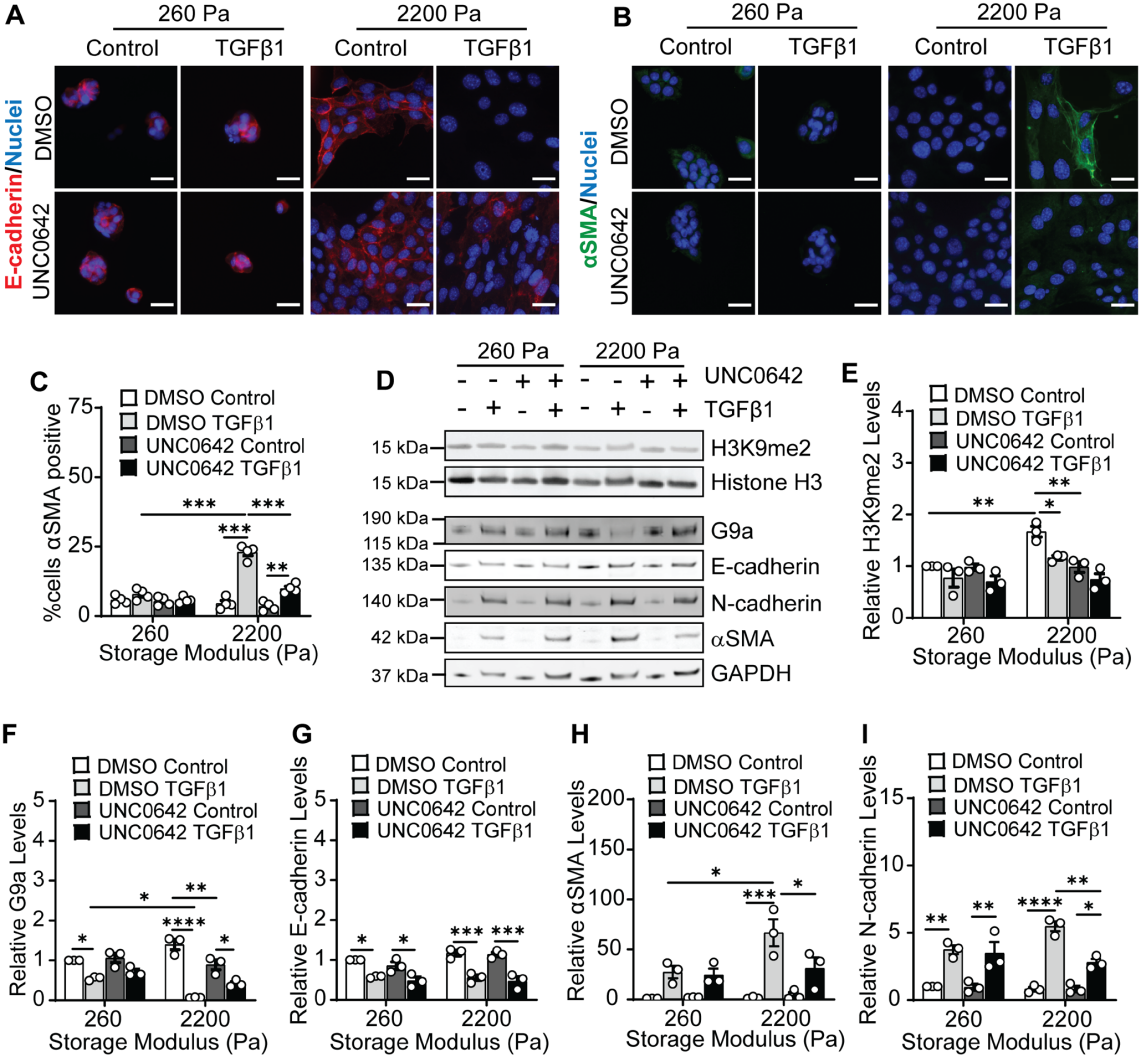
Inhibition of G9a activity impacts H3K9 dimethylation levels and EMT in response to TGFβ1 and matrix stiffness. Immunofluorescence staining for (A) E‐cadherin and (B) αSMA for NMuMG cells cultured on hydrogels and treated with DMSO or the G9a inhibitor UNC0642 (10 nM) with and without TGFβ1 treatment. Scale bars: 25 μm. (C) Quantification of αSMA positive NMuMG cells for various treatment conditions from immunofluorescence staining for αSMA. Data represent mean ± sem for *n* = 4 independent experiments, **p < 0.01, ***p < 0.001. (D) Western blots for H3K9me2, G9a, E‐cadherin, N‐cadherin, and αSMA in NMuMG cells. Densitometric quantification of the relative expression of (E) H3K9me2, (F) G9a, (G) E‐cadherin, (H) αSMA, and (I) N‐cadherin from western blots shown in panel (D). Data are normalized with respect to the soft hydrogel DMSO control sample. Data represent mean ± sem for *n* = 3 independent experiments; *p < 0.05, **p < 0.01, ***p < 0.001, ****p < 0.0001.

Immunofluorescence staining was utilized to examine the expression of the epithelial marker E‐cadherin and the mesenchymal marker αSMA in response to TGFβ1, matrix stiffness, and G9a inhibition (Figure 2A,B). Quantification of the percentage of cells staining positive for αSMA revealed that in cells cultured on stiff hydrogels, treatment with UNC0642 reduces the percentage of αSMA positive cells when cells are treated with TGFβ1 in comparison to treatment with TGFβ1 and DMSO control vehicle (Figure 2C). Vehicle control treated cells cultured on stiff hydrogels show E‐cadherin localization at cell–cell junctions (Figure 2A). TGFβ1 treatment leads to loss of E‐cadherin expression in comparison to the control vehicle treated cells, and treatment with UNC0642 does not restore E‐cadherin expression or localization to cell–cell junctions (Figure 2A).

Western blotting experiments were performed to monitor the effect of UNC0642 on the levels of H3K9me2, G9a, and EMT markers in NMuMG cells (Figure 2D–I). Treatment with UNC0642 decreases H3K9me2 levels in control vehicle treated cells cultured on stiff hydrogels in comparison to DMSO treated cells cultured on stiff hydrogels (Figure 2D,E). When cells are cultured on stiff hydrogels, treatment with UNC0642 promotes a further reduction in the levels of H3K9me2 in TGFβ1 treated cells compared to the DMSO treated cells; however, this reduction in levels is not found to be statistically significant. For cells cultured on soft hydrogels, treatment with UNC0642 does not have a significant effect on the levels of H3K9me2 (Figure 2D,E). Quantification of the relative levels of H3K9me2 from immunofluorescence staining shows consistent findings to observations from western blotting (Figure S3C,D).

Densitometric quantification of western blots for EMT markers revealed that treatment with TGFβ1 results in a decrease in the expression of E‐cadherin in comparison to vehicle treatment in cells cultured on both soft and stiff hydrogels, and treatment with UNC0642 does not restore E‐cadherin levels when cells are also treated with TGFβ1 (Figure 2D,G). In addition, TGFβ1 treatment increases αSMA and N‐cadherin levels when cells are cultured on stiff hydrogels (Figure 2D,H,I), and treatment with UNC0642 attenuates this increase in αSMA and N‐cadherin levels. qRT‐PCR revealed similar trends in the mRNA levels of EMT markers (Figure S3E,F). Together, these observations suggest that inhibition of G9a activity impacts TGFβ1‐induced changes in cell aspect ratio and expression of mesenchymal markers (αSMA and N‐cadherin) when cells are cultured on stiff hydrogels but not on soft hydrogels.

### 3.3 Knockdown of G9a Reduces TGFβ1‐Induced Changes in Cell Morphology and Mesenchymal Marker Expression in Cells Cultured on Stiff Hydrogels

To determine if G9a expression impacts phenotypic changes in cells in response to TGFβ1 when cells are cultured on stiff hydrogels, we depleted G9a levels using siRNA. Transfection with siRNA targeting G9a (siG9a#2) results in more than an 80% reduction in G9a expression in NMuMG cells in comparison to cells transfected with non‐targeting control (NTC) siRNA (Figure S4A–C). NTC siRNA‐transfected cells cultured on stiff hydrogels show an increase in cell spread area and aspect ratio following TGFβ1 treatment in comparison to cells treated with the vehicle control (Figure S4D,E). Knockdown of G9a attenuates this increase in cell spreading and elongation in TGFβ1‐treated cells cultured on stiff hydrogels.

**FIGURE 3:**
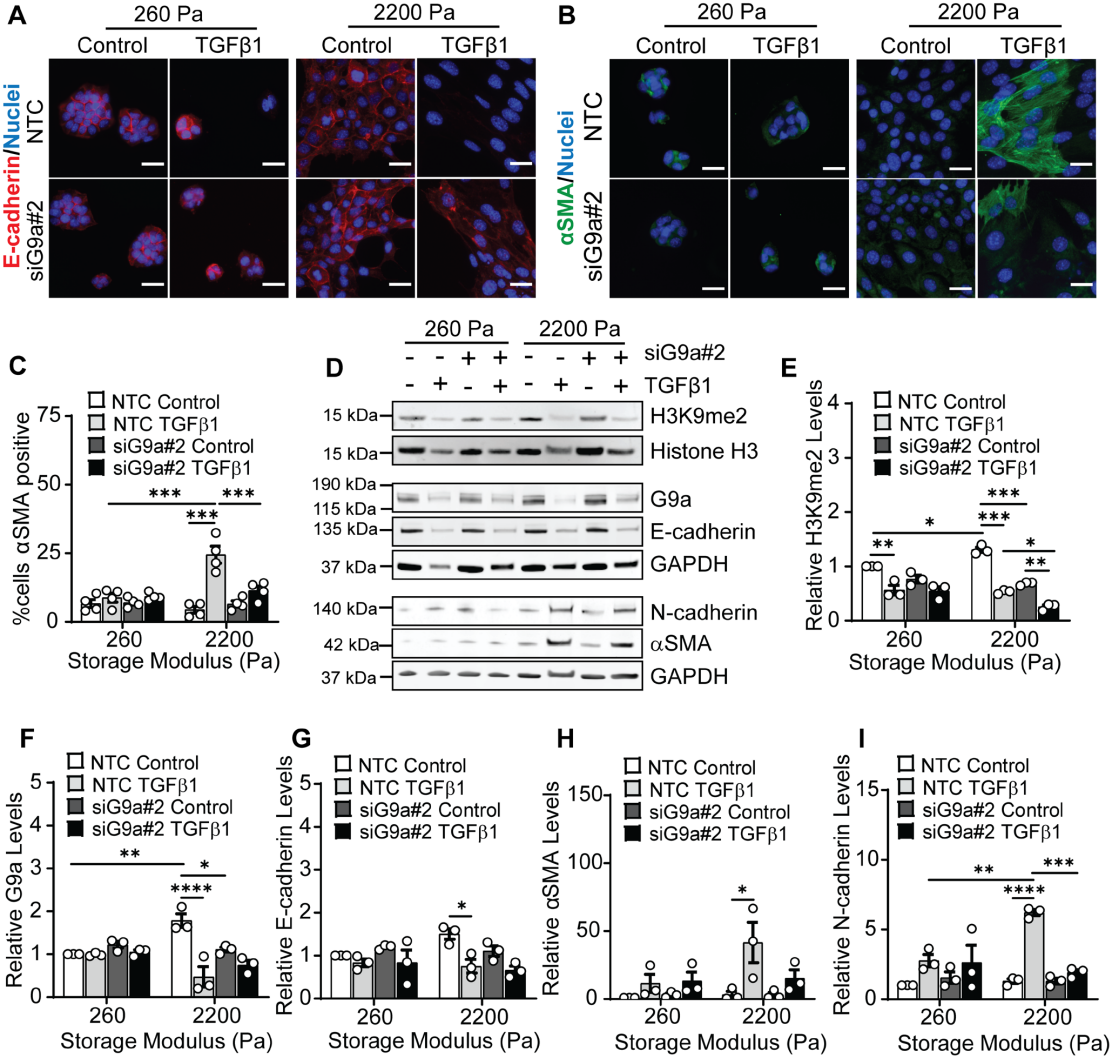
siRNA knockdown of G9a attenuates TGFβ1‐induced changes in H3K9me2, αSMA, and N‐cadherin levels as a function of matrix stiffness. Immunofluorescence staining for (A) E‐cadherin and (B) αSMA in NMuMG cells transfected with NTC siRNA or siG9a#2 with and without TGFβ1 treatment. Scale bars: 25 μm. (C) Quantification of the percentage of αSMA positive cells for various treatment conditions. Data represent mean ± sem for *n* = 4 independent experiments, ***p < 0.001. (D) Western blot for H3K9me2, G9a, E‐cadherin, N‐cadherin, and αSMA in NMuMG cells transfected with NTC siRNA or siG9a#2 with and without TGFβ1 treatment. Densitometric quantification of the relative levels of (E) H3K9me2, (F) G9a, (G) E‐cadherin, (H) αSMA, and (I) N‐cadherin from blots shown in panel (D). Data are normalized with respect to the soft NTC siRNA control sample. Data represent mean ± sem for *n* = 3 independent experiments; *p < 0.05, **p < 0.01, ***p < 0.001, ****p < 0.0001.

Immunofluorescence staining revealed that knockdown of G9a does not affect E‐cadherin cell junction localization as the NTC and siG9a transfected cells cultured on stiff hydrogels show diminished E‐cadherin levels with TGFβ1 treatment (Figure 3A). Knockdown of G9a decreases the percentage of TGFβ1‐treated cells staining positive for αSMA when cultured on stiff hydrogels in comparison to the NTC‐transfected TGFβ1‐treated cells (Figure 3B,C; Figure S4F). Western blotting experiments revealed that knockdown of G9a significantly reduces H3K9me2 levels in both the control vehicle and TGFβ1‐treated cells cultured on stiff hydrogels in comparison to the NTC‐transfected cells (Figure 3D,E). A reduction in G9a levels does not significantly impact H3K9me2 levels in cells cultured on soft hydrogels. Western blots for EMT markers are consistent with immunofluorescence staining and reveal that G9a knockdown does not affect E‐cadherin levels; TGFβ1 treatment promotes a decrease in E‐cadherin in both the NTC and siG9a transfected cells (Figure 3D,G). Western blotting also revealed that in cells cultured on stiff hydrogels, knockdown of G9a reduces TGFβ1‐induced αSMA and N‐cadherin expression in comparison to the NTC siRNA transfected cells treated with TGFβ1 (Figure 3D,H,I). These results are consistent with G9a inhibition results and indicate that G9a can regulate cell morphology and the expression of mesenchymal markers in mammary epithelial cells in response to TGFβ1 when cells are cultured on stiff hydrogels.

### 3.4 Knockdown of G9a Reduces YAP Nuclear Localization in Response to TGFβ1 and Matrix Stiffness

**FIGURE 4:**
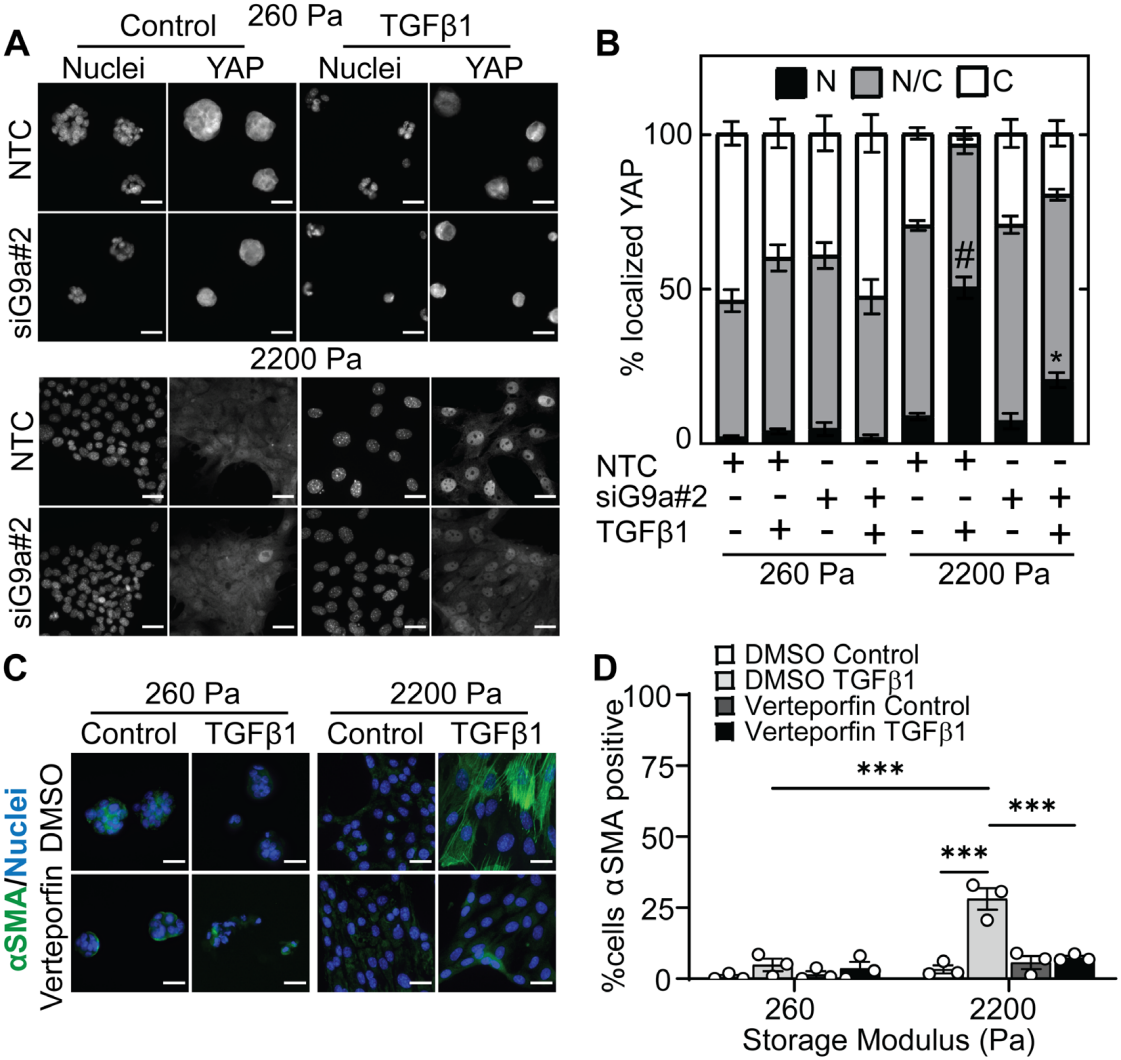
Knockdown of G9a impacts YAP subcellular localization and inhibiting YAP attenuates TGFβ1‐induced αSMA expression and cell morphology changes as a function of matrix stiffness. (A) Immunofluorescence staining for YAP in NMuMG cells transfected with NTC siRNA or siG9a#2 with and without TGFβ1 treatment. Scale bars: 25 μm. (B) Quantification of the percentage of cells with nuclear (N), pancellular (N/C), or cytoplasmic (C) YAP localization under different treatment conditions. Data represent mean ± sem for *n* = 3; #*p* < 0.001 with respect to all other samples, *p < 0.05 with respect to the stiff hydrogel siG9a control and soft hydrogel siG9a TGFβ1 samples. (C) Immunofluorescence staining for αSMA in NMuMG cells cultured on hydrogels and treated with DMSO or YAP inhibitor Verteporfin (4 μM) with and without TGFβ1 treatment. Scale bars: 25 μm. (D) Quantification of αSMA positive NMuMG cells cultured on soft and stiff hydrogels and treated with DMSO or Verteporfin in the presence and absence of TGFβ1. Data represent mean ± sem for *n* = 3 independent experiments; ***p < 0.001.

YAP is a mechanoresponsive protein [53] that regulates the expression of αSMA [30]. Previous studies have shown that TGFβ1 enhances nuclear localization of YAP in NMuMG cells [54]. We hypothesized that the reduction in αSMA expression following G9a inhibition and knockdown in TGFβ1‐treated NMuMG cells cultured on stiff hydrogels may be regulated by YAP signaling. To test this hypothesis, we examined the subcellular localization of YAP via immunofluorescence staining. Cells cultured on stiff hydrogels exhibit enhanced nuclear localization of YAP in comparison to cells cultured on soft hydrogels (Figure S5). YAP nuclear localization is further elevated in cells cultured on stiff hydrogels following TGFβ1 treatment. Knockdown of G9a attenuates the increase in the percentage of cells with nuclear localized YAP when cells are cultured on stiff hydrogels and treated with TGFβ1 (Figure 4A,B).

To confirm the role of YAP in regulating αSMA expression, we treated the cells with the YAP inhibitor Verteporfin. Immunofluorescence staining revealed that treatment with Verteporfin decreases the percentage of TGFβ1‐treated cells expressing αSMA as compared to the TGFβ1/DMSO‐treated cells when cultured on stiff hydrogels (Figure 4C,D). For cells cultured on stiff hydrogels, Verteporfin also reduces TGFβ1‐induced cell spreading but does not affect cell aspect ratio (Figure S6A,B). These observations suggest that the subcellular localization of YAP is regulated by TGFβ1, G9a, and matrix stiffness and that YAP plays an important role in the regulation of αSMA expression downstream of TGFβ1. Furthermore, knockdown of G9a leads to an increase in cytoplasmic localization of YAP, which may provide a mechanistic connection between G9a inhibition and knockdown leading to a reduction in αSMA expression in TGFβ1‐treated cells cultured on stiff hydrogels.

### 3.5 Inhibition of LATS Kinases Increases YAP Nuclear Localization and αSMA Expression in TGFβ1‐Treated Mammary Epithelial Cells Cultured on Stiff Hydrogels

**FIGURE 5:**
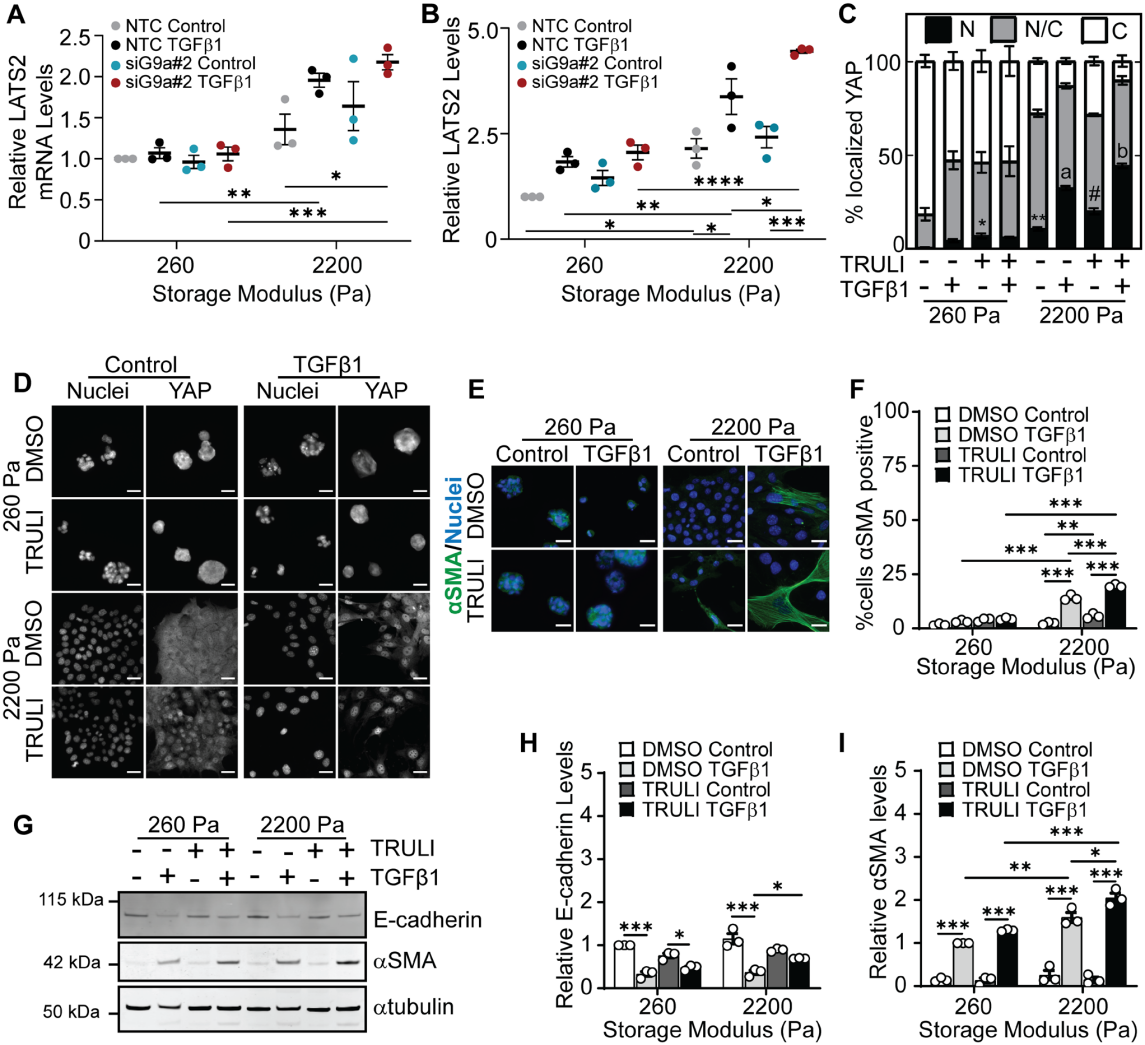
G9a regulates LATS kinase which acts upstream of YAP and αSMA. (A) Quantitative real‐time PCR for LATS2 and (B) relative levels of LATS2 quantified from immunofluorescence staining in NMuMG cells cultured on hydrogels with storage moduli of 260 Pa and 2200 Pa and transfected with non‐targeting control siRNA (NTC) or siRNA targeting G9a (siG9a#2) with and without TGFβ1 treatment. Data represent mean ± sem for *n* = 3 independent experiments; *p < 0.05, **p < 0.01, ***p < 0.001, ****p < 0.0001. (C) Quantification of the percentage of cells with nuclear (N), pancellular (N/C), or cytoplasmic (C) YAP localization in cells following treatment with DMSO or LATS1/2 kinase inhibitor TRULI (15 μM) with and without TGFβ1 treatment. Data represent mean ± sem for *n* = 3 independent experiments; *p < 0.05, **p < 0.01 with respect to soft DMSO control sample, #*p* < 0.01 with respect to stiff DMSO control sample, ^a^ *p* < 0.001 with respect to all other samples except stiff TRULI TGFβ1, ^b^ *p* < 0.001 with respect to all other samples. Immunofluorescence staining for (D) YAP and (E) αSMA in NMuMG cells under different treatment conditions. Scale bars: 25 μm. (F) Quantification of αSMA positive NMuMG cells treated with DMSO or TRULI in the presence and absence of TGFβ1. Data represent mean ± sem for *n* = 3 independent experiments; **p < 0.01, ***p < 0.001. (G) Western blot for E‐cadherin and αSMA in NMuMG cells cultured on hydrogels and treated with DMSO or TRULI with and without treatment with TGFβ1. Densitometric quantification of the relative expression of (H) E‐cadherin and (I) αSMA from western blots shown in panel (G). Data are normalized with respect to the soft DMSO control sample. Data represent mean ± sem for *n* = 3 independent experiments; *p < 0.05, **p < 0.01, ***p < 0.001.

YAP is a member of the Hippo signaling pathway [55, 56] and LATS kinases can regulate the phosphorylation state and subcellular localization of YAP [57, 58]. Indeed, phosphorylation of YAP by LATS kinases leads to retention of YAP within the cytoplasm [58, 59]. As such, we hypothesized that G9a may regulate αSMA expression via repression of LATS kinases. Quantitative real‐time PCR was performed to examine the mRNA levels of LATS1 and LATS2 in NMuMG cells transfected with NTC or G9a siRNA in response to TGFβ1 and matrix stiffness. LATS2 mRNA expression was significantly higher in TGFβ1 treated cells cultured on stiff hydrogels in comparison to when cultured on soft hydrogels (Figure 5A). Knockdown of G9a elevates the expression of LATS2 in both the control and TGFβ1‐treated cells when cultured on stiff hydrogels in comparison to the NTC siRNA‐transfected cells (Figure 5A). For cells cultured on soft hydrogels, knockdown of G9a does not impact the levels of LATS2. LATS1 mRNA expression remains nearly constant across treatment conditions (Figure S7A). Quantification of relative LATS2 levels from immunofluorescence staining revealed an increase in LATS2 levels in cells transfected with siRNA targeting G9a and treated with TGFβ1 in comparison to cells treated with control vehicle when cultured on stiff hydrogels (Figure 5B, Figure S7B,C). In NTC siRNA‐transfected cells cultured on stiff matrix, the increase in LATS2 expression following TGFβ1 treatment correlates with an observed decrease in G9a expression (Figure 1F).

**FIGURE 6:**
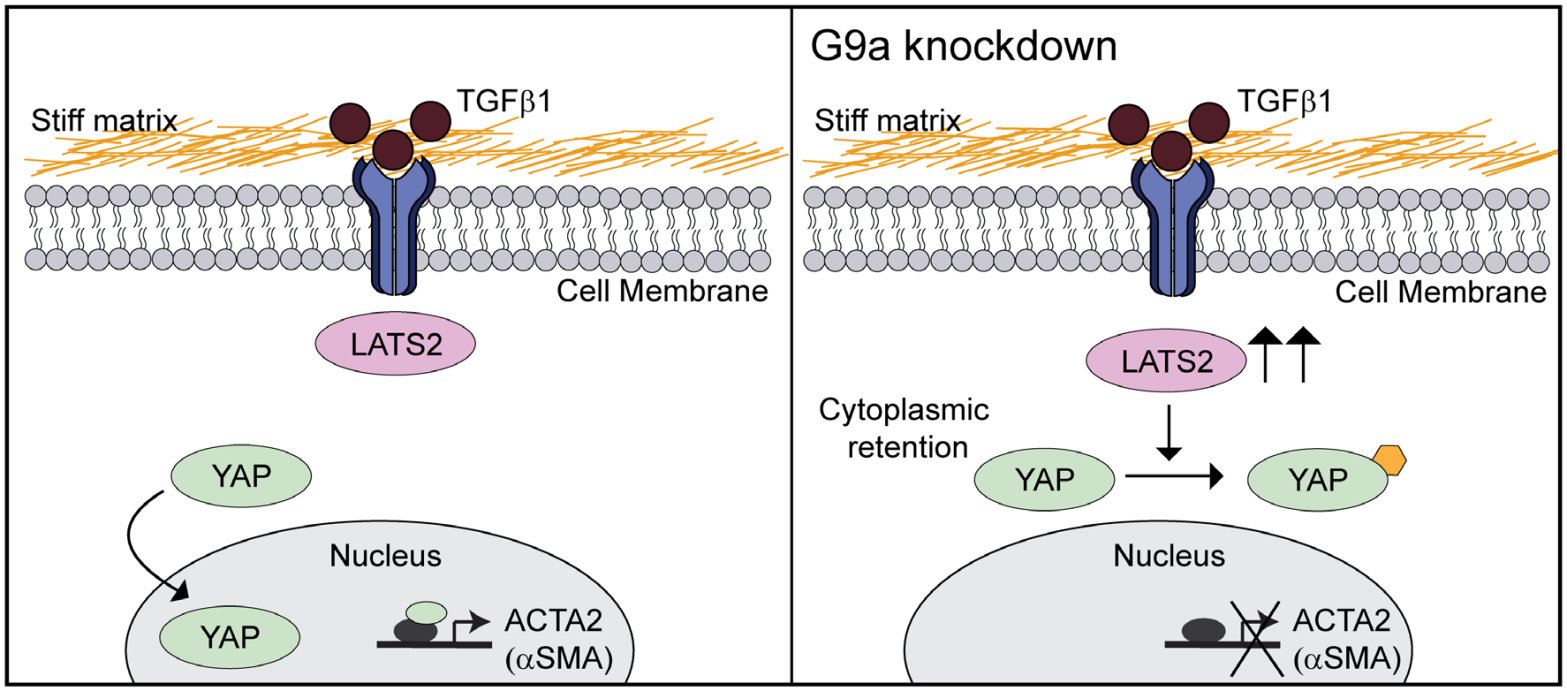
Knockdown of G9a promotes an increase in LATS2 expression, cytoplasmic retention of YAP, and a decrease in αSMA expression in TGFβ1‐treated cells cultured on stiff substrata.

To elucidate whether the activity of LATS kinases regulates αSMA expression in TGFβ1‐treated cells cultured on stiff hydrogels, we used a small molecule LATS kinase inhibitor TRULI [60]. Western blotting confirmed that treatment with TRULI reduced phosphorylation of YAP (Figure S8). Immunofluorescence staining revealed that the percentage of cells with nuclear localized YAP increases in cells cultured on stiff hydrogels following treatment with TGFβ1 and TRULI in comparison to cells treated with TGFβ1 alone (Figure 5C,D). Furthermore, when cells are cultured on stiff hydrogels, treatment with TRULI increases the percentage of TGFβ1‐treated cells that stain positive for αSMA in comparison to treatment with TGFβ1 and DMSO (Figure 5E,F). We also performed western blotting to examine the levels of E‐cadherin and αSMA following TRULI treatment. E‐cadherin and αSMA levels increase in TGFβ1 treated cells cultured on stiff hydrogels following treatment with TRULI (Figure 5G–I). TRULI treatment does not significantly affect E‐cadherin and αSMA levels in cells cultured on soft hydrogels. Furthermore, examination of cell morphology revealed that treatment with TRULI elevates TGFβ1‐induced cell spreading but does not affect cell aspect ratio when cells are cultured on stiff hydrogels (Figure S6C,D). These findings suggest that LATS kinase inhibition promotes YAP nuclear localization and, in turn, an increase in the expression level of αSMA in response to TGFβ1 when cells are cultured on stiff hydrogels. Furthermore, this data suggests a mechanism whereby αSMA expression is regulated by a G9a‐LATS‐YAP signaling cascade in cells cultured on stiff hydrogels (Figure 6).

## 4 Discussion

TGFβ and G9a contribute to fibrosis and cancer, and some studies have shown that TGFβ1 promotes the expression of G9a in rat kidney epithelial cells and human peritoneal mesothelial cells [61, 62]. Methylation of H3K9, which is mediated in part by G9a, is enhanced in melanoma cells and in lung fibroblasts when cultured on stiff matrices in comparison to on softer matrices [17, 63]. In addition, treatment with TGFβ1 amplifies H3K9 methylation in fibroblasts [17]. In contrast to these previous findings, we find that TGFβ1 treatment significantly decreases the levels of G9a in mammary epithelial cells cultured on stiff hydrogels that mimic the mechanical properties of breast tumors. Furthermore, we find that inhibition and knockdown of G9a further reduce H3K9me2 levels in cells cultured on stiff hydrogels. Discrepancies in the regulation of G9a and H3K9 methylation levels by TGFβ1 between our study and others may be due to cell type, matrix composition and stiffness, or to the differential regulation of the mono‐, di‐, and trimethylation of H3K9. Overall, our observations provide new information about the combined effects of growth factor signaling and mechanical cues on the regulation of histone modifications and modification enzymes. Future studies focused on examination of the impact of mechanical signals on other histone modification enzymes are warranted.

While some studies support a role for G9a in the regulation of the repression of E‐cadherin [22, 23, 24, 64], other studies find that the inhibition of G9a does not impact E‐cadherin expression in other contexts [62, 65]. These previous findings suggest that the regulatory effect of G9a on E‐cadherin might be cell type and context dependent. In this work, we find that inhibiting G9a activity by treatment with UNC0642 did not restore E‐cadherin levels in TGFβ1‐treated cells cultured on stiff hydrogels compared to the DMSO‐treated cells. In addition, knockdown of G9a expression did not impact E‐cadherin levels in TGFβ1‐treated cells cultured on both the soft and stiff hydrogels in comparison to NTC siRNA‐transfected cells. Further studies are needed to clarify the regulation of E‐cadherin by G9a for different cell types, growth factor stimulation conditions, and cellular microenvironments.

Matrix stiffness has been shown to regulate TGFβ1‐induced expression of mesenchymal markers including N‐cadherin and αSMA, with cells cultured on stiff hydrogels exhibiting increased expression of these markers in comparison to cells cultured on soft hydrogels [7, 9]. Consistent with these previous reports, we find that N‐cadherin and αSMA protein expression is significantly increased in TGFβ1‐treated cells cultured on stiff hydrogels. Previous reports have also demonstrated a role for G9a in regulating the expression of αSMA. Treatment with BIX01294, a G9a inhibitor, reduces αSMA expression in a model of unilateral ureteral obstruction in mice [62] and decreases the levels of myofibroblasts expressing αSMA in methylglyoxal‐treated mice [61]. In addition, BIX01294 treatment attenuates TGFβ1‐induced αSMA and fibronectin expression in human peritoneal mesothelial cells [61] and promotes a decrease in matrix stiffness‐induced αSMA expression in human lung fibroblasts [17]. In this work, in agreement with the findings reported in the abovementioned studies, we show that inhibition and knockdown of G9a attenuate TGFβ1‐induced αSMA expression in mammary epithelial cells cultured on stiff hydrogels. A small percentage of siG9a‐transfected cells cultured on stiff hydrogels show expression of αSMA following treatment with TGFβ1 (Figure 3B,C); this could arise from heterogeneity in knockdown of G9a within the cell population (Figure S4A–C). Future examination of heterogeneity in cell response to TGFβ1 could provide further insight into mechanisms governing epithelial‐mesenchymal plasticity. Overall, these findings suggest that matrix stiffness and G9a coordinate to regulate αSMA expression in response to TGFβ1 stimulation in mammary epithelial cells.

Previous studies suggest that the transcription factor YAP regulates αSMA expression. Overexpression of YAP elevates αSMA mRNA and protein levels in neonatal rat cardiac fibroblasts [30]. Additionally, application of strain to human scleral fibroblasts promotes an increase in αSMA expression, and knockdown or inhibition of YAP attenuates strain‐induced αSMA expression [66]. Furthermore, in fibrotic tissue samples which exhibit higher stiffness than normal tissues, αSMA positive myofibroblasts show YAP localization in the nucleus [67]. In this study, we find that inhibition of YAP activity using Verteporfin leads to attenuation of TGFβ1‐induced αSMA expression in cells cultured on stiff hydrogels. These data suggest an important role for YAP in mediating TGFβ1‐induced αSMA expression in epithelial cells cultured in stiff microenvironments.

LATS kinases, components of the Hippo signaling pathway, can regulate YAP phosphorylation and impact YAP subcellular localization and signal transduction. Phosphorylation at serine 127 leads to the binding of YAP to the protein 14‐3‐3 and cytoplasmic localization, while phosphorylation at serine 381 is associated with the ubiquitination of YAP, which promotes its degradation [58, 59]. In human cholangiocarcinoma cells, G9a and H3K9me2 levels are increased at the promoter region of the LATS2 gene, which promotes its repression, while G9a knockdown increases LATS2 expression and reduces YAP nuclear localization [33]. Consistent with this study, we find that the knockdown of G9a results in an increase in the mRNA and protein levels of LATS2, a reduction in the nuclear localization of YAP, and a decrease in the expression of αSMA in TGFβ1‐treated cells cultured on stiff hydrogels, but not in cells cultured on soft hydrogels. In addition, the subcellular localization of YAP can be regulated by mechanical cues [28, 29] and by PI3K signaling [68, 69]. In mammary epithelial cells and breast cancer cells, PI3K and phosphoinositide‐dependent kinase (PDK1) act as upstream negative regulators of LATS1/2, thereby promoting YAP nuclear localization [68, 69], and PI3K inhibitors block YAP nuclear localization [68]. Here, we observe that TGFβ1‐treated NTC siRNA‐transfected cells cultured on stiff hydrogels show increased YAP nuclear localization, which is consistent with previously reported activation of PI3K/Akt signaling in TGFβ1‐treated mammary epithelial cells cultured on stiff hydrogels [9]. In rhabdomyosarcoma cells, inhibition and knockdown of G9a reduce the activation of Akt, which is a downstream effector of PI3K [70]; however, the impact of G9a on PI3K/Akt signaling in response to TGFβ1 has not been elucidated. Further experiments examining the interplay of G9a, PI3K/Akt, and the Hippo signaling pathway in response to matrix stiffness and growth factor stimulation are warranted and may provide additional insight into how microenvironmental mechanical properties regulate TGFβ1‐induced gene expression and epithelial‐mesenchymal plasticity.

## Author Contributions

Chinmay S. Sankhe, Jessica L. Sacco, Joy Kirigo, Thomas K. Wood, and Esther W. Gomez conceptualized and designed the experiments. Chinmay S. Sankhe, Jessica L. Sacco, Victoria L. Crunkleton, Malcom Díaz García, Matthew J. Bierowski, David Vidotto Rezende Soares, Jacob A. Karnick, Rachel L. Cecco, Arefeh Abbasi, and Joy Kirigo performed the experiments. Chinmay S. Sankhe, Jessica L. Sacco, and Esther W. Gomez wrote the manuscript. All authors contributed to data analysis and provided comments regarding the manuscript. All authors have approved the manuscript.

## Conflicts of Interest

The authors declare no conflicts of interest.

## Supporting information


**Data S1.** Supporting Information.

## Data Availability

The data that support the findings of this study are available in the methods and/or supporting information—S1 of this article.
