# Supplementary material for: Matrix Stiffness Regulates TGFβ1‐Induced αSMA Expression via a G9a‐LATS‐YAP Signaling Cascade
Source: FASEB Bioadv. 2025 Jul 14;7(7):e70035. doi: 10.1096/fba.2025-00117 (PMC12256985; doi:10.1096/fba.2025-00117)
Supplement: Supplementary file 1 — Data S1. Supporting Information. [file FBA2-7-e70035-s001.pdf]

Supplemental Information for

Matrix stiffness regulates TGFβ1-induced αSMA expression via a G9a-LATS-YAP signaling cascade

Chinmay S. Sankhe[1†], Jessica L. Sacco[1†], Victoria L. Crunkleton[2], Malcom Díaz García[3], Matthew J. Bierowski[1,4], David Vidotto Rezende Soares[1], Jacob A. Karnick[1], Rachel L. Cecco[1], Arefeh Abbasi[1], Joy Kirigo[1], Thomas K. Wood[1], Esther W. Gomez[1,5,*]

[1] Department of Chemical Engineering, Pennsylvania State University, University Park, PA 16802 USA

[2] Department of Materials Science and Engineering, North Carolina State University, Raleigh, NC 27695 USA

[3] Department of Mechanical Engineering, University of Puerto Rico, Mayagüez 00681 PR

[4] Penn State College of Medicine, Pennsylvania State University, Hershey, PA 17033 USA

[5] Department of Biomedical Engineering, Pennsylvania State University, University Park, PA 16802 USA

[†]Authors contributed equally to this work

[*]Corresponding author: ewg10@psu.edu

Table S1. Forward and reverse primers used for quantitative real-time PCR

Marker	Forward Primer	Reverse Primer
G9a	ACCACCTGTGTGACCCCAACATCA	ATCGGTCACCGTAGTCAAAGCCCA
LATS1	AGCAGCACGTAGAGAACGTC	TCTCATTTGATCCTGGGCATCT
LATS2	TGCACTGGATTCAGGTGGACTCA	GAGAATGTGCCAGGCACCTCT
E-cadherin	CAACGAATCCCTCAAAGACC	GATAGAGAAGCCATTGAAAA
αSMA	GGCACCACTGAACCCTAAG	CCAGAGTCCAGCACAATACC
GAPDH	ACTGGCATGGCCTTCCGTGTTCCTA	TCAGTGTAGCCCAAGATGCCCTTC

Table S2. Composition and mechanical properties of polyacrylamide hydrogels. Young's modulus is reported based on using a Poisson's ratio of 0.45.

	**Acrylamide (v/v %)**	**Bis-acryamide (v/v %)**	**APS (w/v %)**	**TEMED (v/v %)**	**Storage modulus (G'; Pa)**	**Young's modulus (E; Pa)**
Soft	5	0.015	0.05	0.05	260 ± 30	750 ± 60
Stiff	7.5	0.2	0.05	0.05	2200 ± 70	6400 ± 170

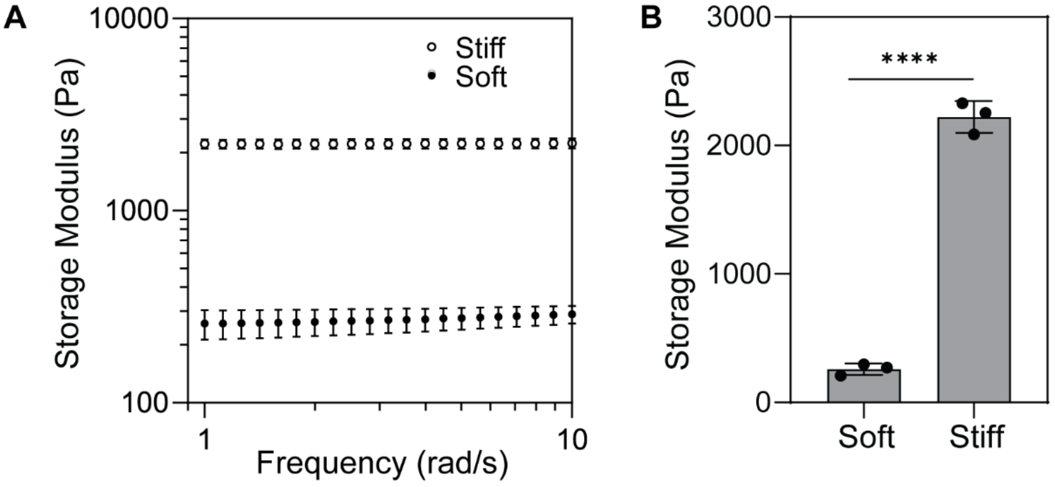

Figure S1. Storage modulus for polyacrylamide hydrogels determined from rheology. (A) Storage modulus (G') as a function of frequency. Frequency sweep performed at 2% strain. (B) Storage modulus measured at a frequency of 1 rad/s. Data represent the mean ± sem for n =3 independent experiments; ****p<0.0001.

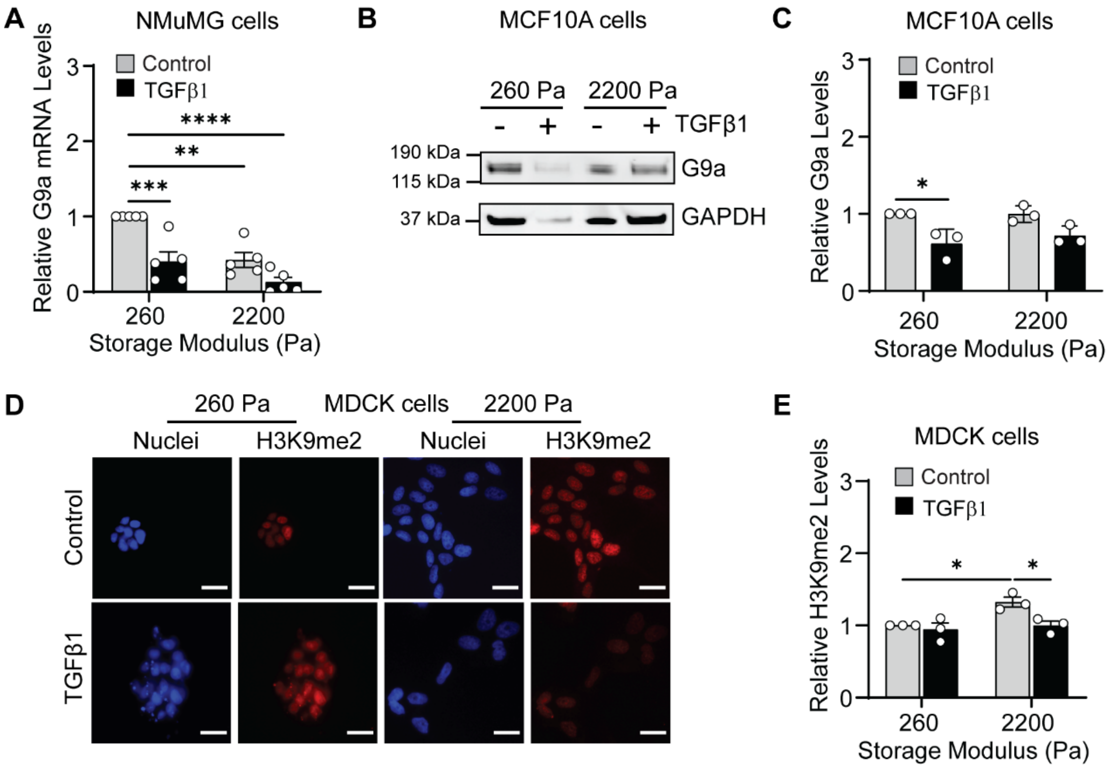

Figure S2. Matrix stiffness regulates G9a and histone lysine 9 dimethylation levels during TGFβ1-induced epithelial mesenchymal transition. (A) Quantitative real-time PCR reveals relative G9a mRNA levels in NMuMG cells as a function of matrix stiffness and TGFβ1 treatment. (B,C) Western blot and densitometric analysis of G9a protein levels in MCF10A mammary epithelial cells as a function of matrix stiffness and TGFβ1 treatment. (D,E) Immunofluorescence staining and quantification of H3K9me2 levels in Madin Darby Canine Kidney (MDCK) epithelial cells cultured on G' = 260 Pa and 2200 Pa hydrogels in the presence and absence of TGFβ1. Scale bars: 25 µm. Data are normalized with respect to the soft hydrogel control sample. Data represent mean ± sem for n=3-5 independent experiments; $^{*}p<0.05$, $^{**}p<0.01$, $^{***}p<0.001$, $^{****}p<0.0001$.

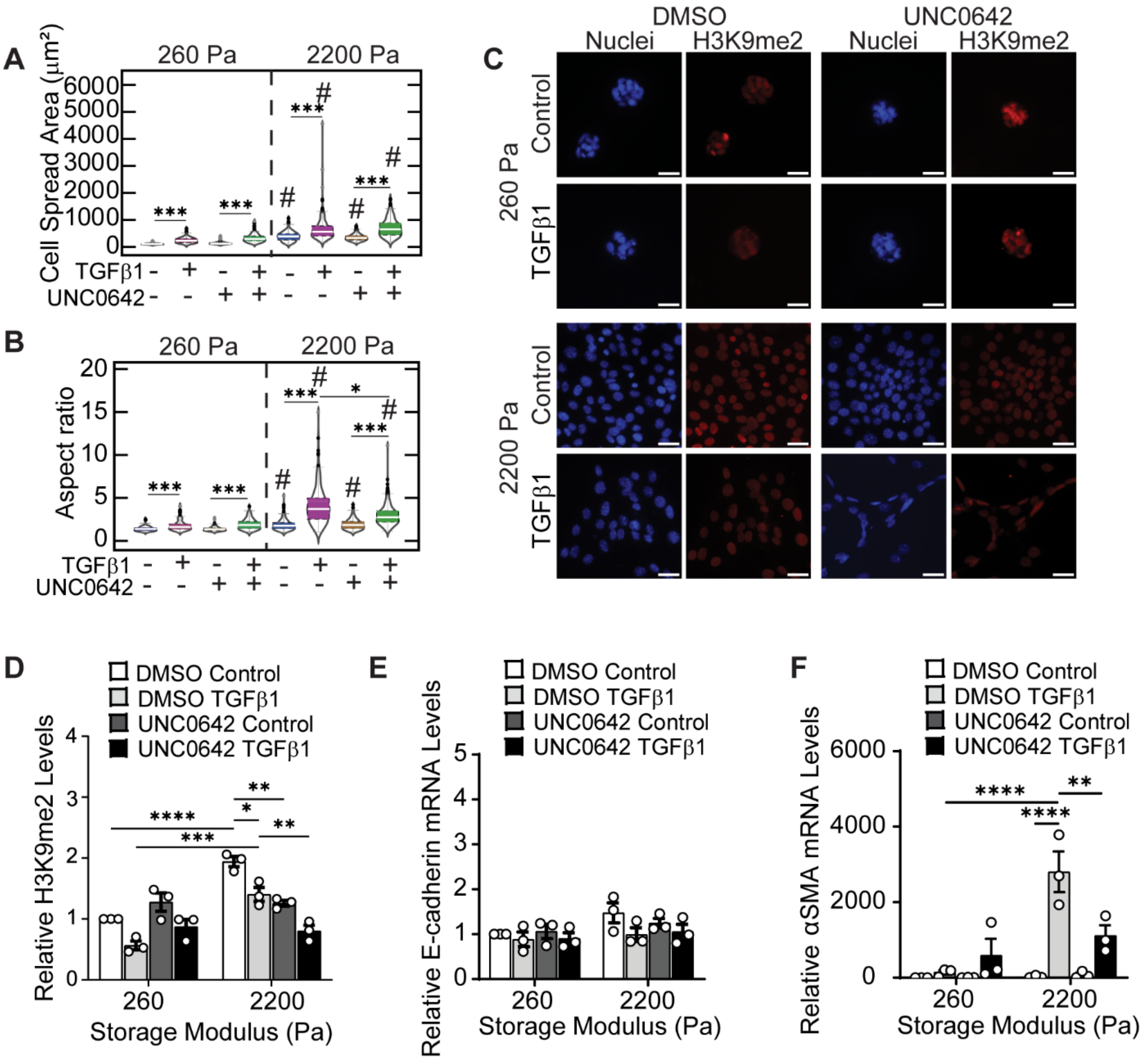

Figure S3. Inhibition of G9a activity impacts cell morphology, H3K9 dimethylation levels and EMT in response to TGFβ1 and matrix stiffness. Violin distribution plot overlaid with box plot of the cell (A) spread area and (B) aspect ratio for NMuMG cells cultured on G' = 260 Pa and 2200 Pa hydrogels and treated with DMSO or the G9a inhibitor UNC0642 (10 nM) with and without treatment with TGFβ1. At least 85 cells were quantified for every treatment condition, $*p<0.05$, $***p<0.001$ as indicated, and $\#p<0.001$ with respect to cells cultured on the soft hydrogels. (C) Immunofluorescence staining for H3K9me2. Scale bars: 25 μm. (D) Quantification of the relative integrated intensity of H3K9me2 from images in panel C. Quantitative RT-PCR analysis of the relative mRNA levels of (E) E-cadherin and (F) αSMA. Data represent mean ± sem for n = 3 independent experiments. $*p<0.05$, $**p<0.01$, $***p<0.001$, $****p<0.0001$.

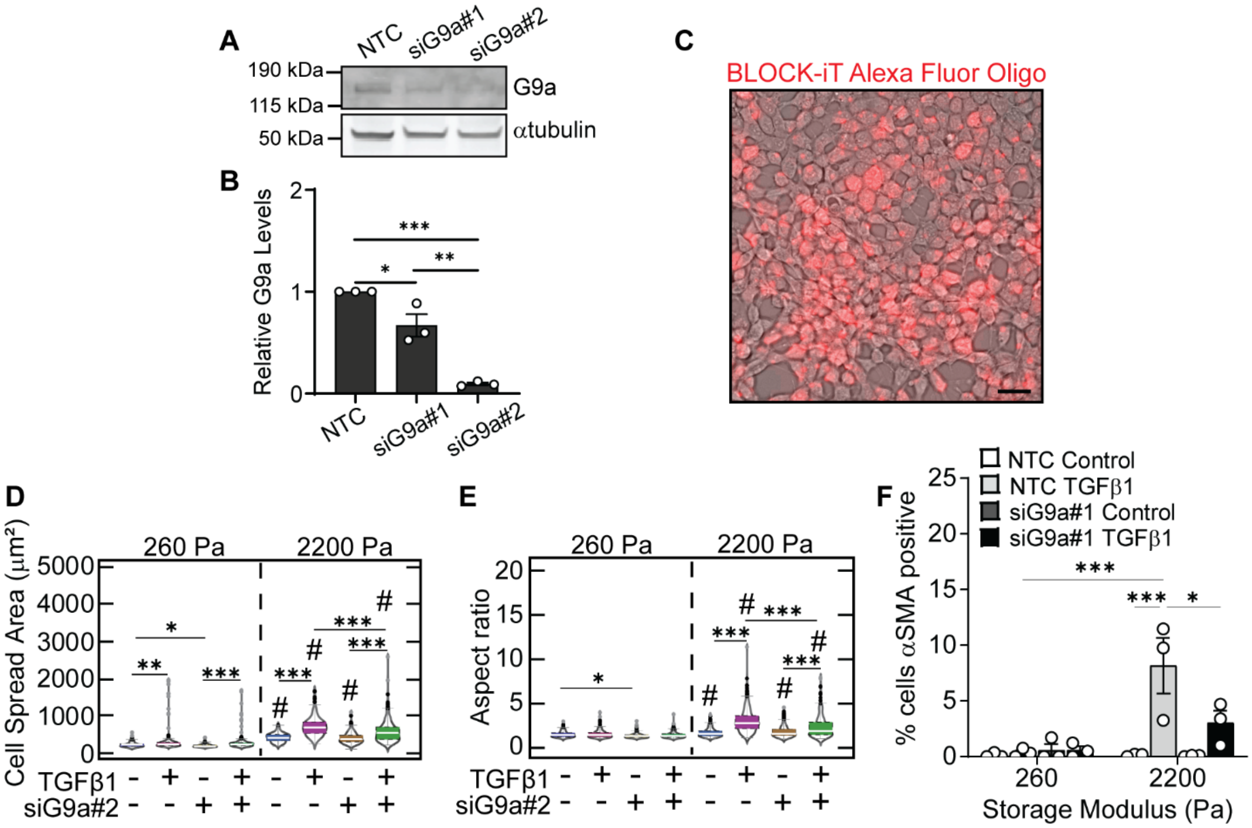

Figure S4. Depletion of G9a levels in NMuMG cells using siRNA knockdown regulates cell morphology. (A) Western blotting and (B) densitometric analysis of G9a levels in NMuMG cells transfected with negative control (NTC) siRNA and two siRNAs targeting G9a. Data are normalized with respect to the NTC siRNA transfected sample. Data represent mean ± sem for n=3 independent experiments; *p<0.05, **p<0.01, ***p<0.001. (C) Overlay of brightfield and fluorescence images showing uptake of BLOCK-iT Alexa Fluor Red Fluorescent Control oligo to examine efficiency of siRNA transfection. Scale bar: 25 µm. (D,E) Violin distribution plot overlaid with box plot of the cell (D) spread area and (E) aspect ratio of NMuMG cells cultured on soft (G' = 260 Pa) and stiff (G' = 2200 Pa) hydrogels and transfected with non-targeting control (NTC) siRNA and G9a siRNA#2 in the presence and absence of TGFβ1. At least 200 cells were quantified for every treatment condition, *p<0.05, **p<0.01, ***p<0.001 as indicated, and #p<0.001 with respect to cells cultured on soft hydrogels. (F) Quantification of the percentage of αSMA positive NMuMG cells transfected with NTC siRNA or siG9a#1 with and without TGFβ1 treatment. Data represent mean ± sem for n=3 independent experiments; *p<0.05, ***p<0.001.

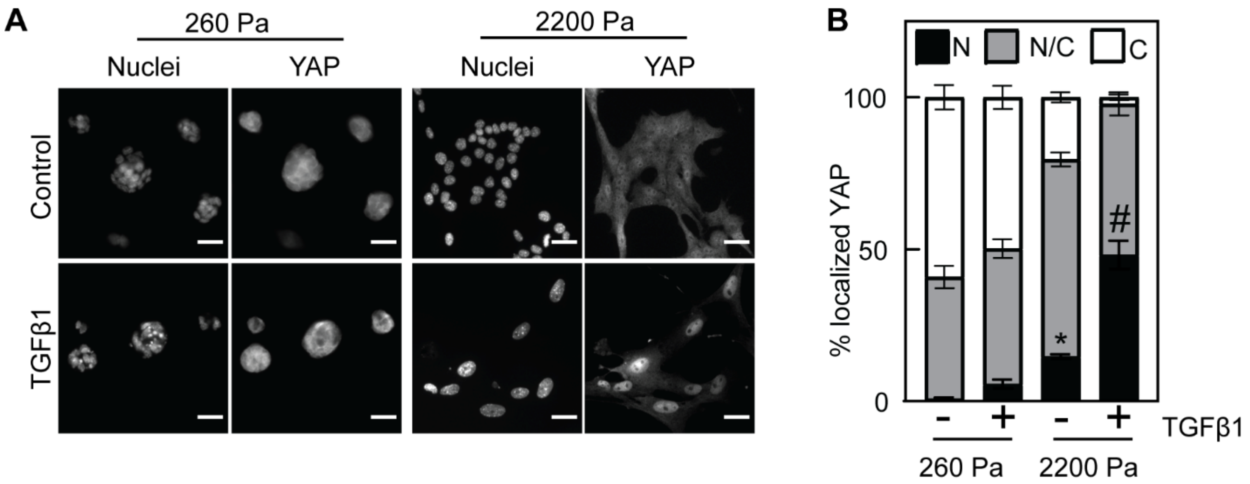

Figure S5. Matrix stiffness and TGFβ1 signaling control the subcellular localization of YAP. (A) Immunofluorescence staining for YAP in NMuMG cells cultured on G' = 260 Pa and 2200 Pa hydrogels in the presence and absence of TGFβ1. Scale bars: 25 μm. (B) Quantification of the percentage of cells with YAP localized to the nucleus (N), pancellular (N/C), or cytoplasm (C) regions as a function of TGFβ1 treatment and matrix stiffness. Data represent mean ± sem for n=3 independent experiments; *$p<0.05$ with respect to nuclear localized YAP in the soft control sample, #$p<0.001$ with respect to nuclear localized YAP in all other samples.

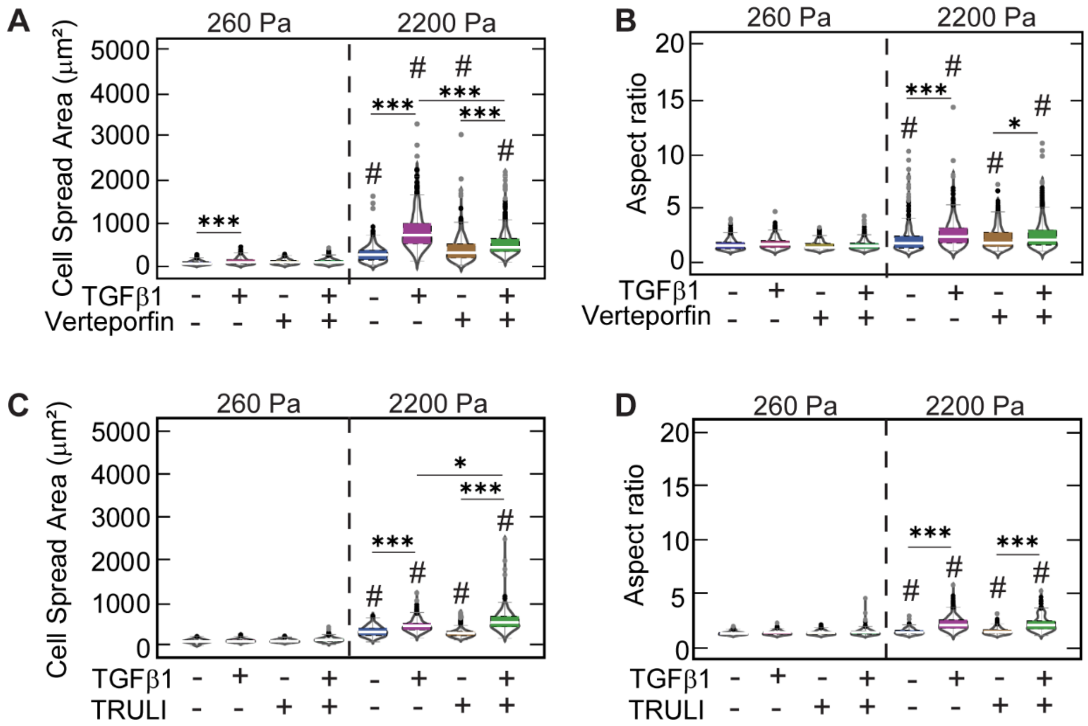

Figure S6. YAP and LATS kinase inhibition impact cell morphology in TGFβ1-treated cells cultured on stiff hydrogels. Violin distribution plot overlaid with box plot of the cell (A,C) spread area and (B,D) aspect ratio for NMuMG cells cultured on soft and stiff hydrogels and treated with (A,B) Verteporfin or (C,D) TRULI in the presence and absence of TGFβ1. At least 160 cells were quantified for every treatment condition, $*p<0.05$, $***p<0.001$ as indicated, and $\#p<0.001$ in comparison to respective soft samples.

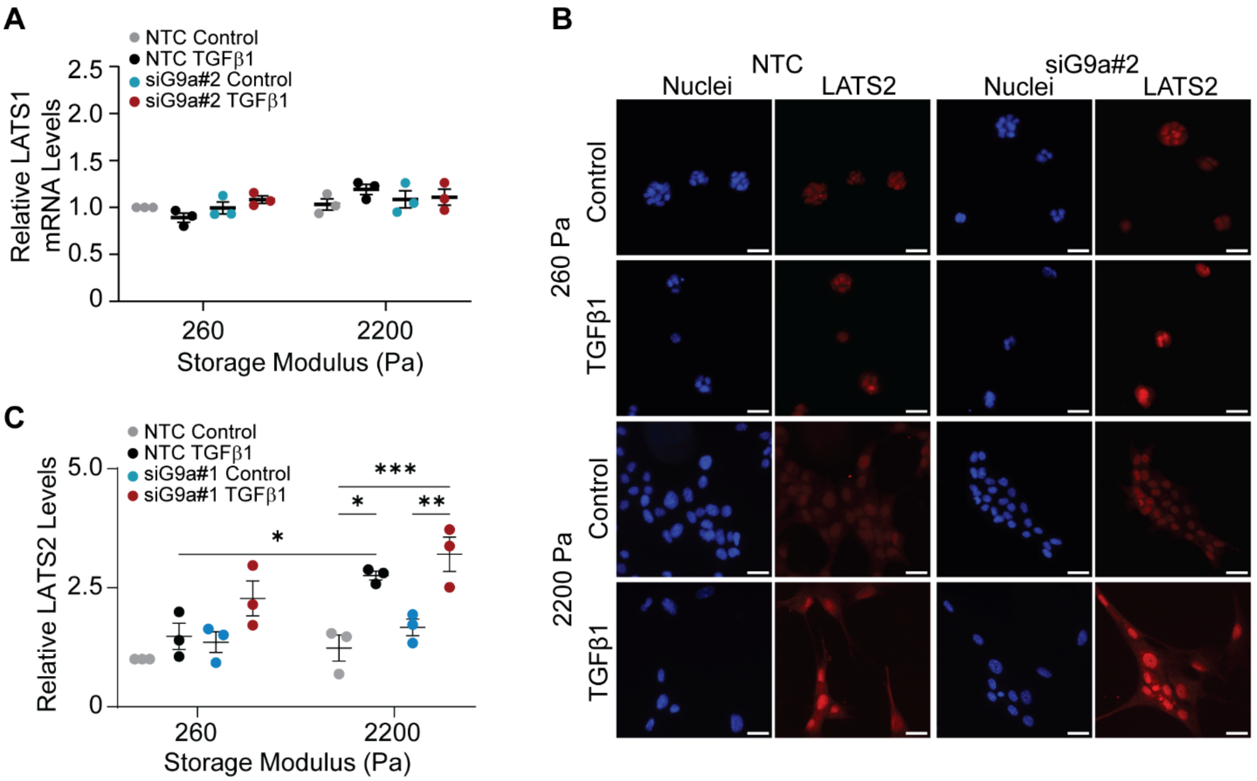

Figure S7. LATS1 and LATS2 expression in response to matrix stiffness and TGFβ1. (A) Quantitative real-time PCR for LATS1 and (B) immunofluorescence staining for LATS2 in NMuMG cells cultured on hydrogels with storage moduli of 260 Pa and 2200 Pa and transfected with non-targeting control siRNA (NTC) or siRNA targeting G9a (siG9a#2) with and without TGFβ1 treatment. Scale bars: 25 µm. (C) Relative LATS2 levels quantified from immunofluorescence images of NMuMG cells transfected with non-targeting control siRNA (NTC) or siRNA targeting G9a (siG9a#1), cultured on hydrogels with storage moduli of 260 Pa and 2200 Pa, and treated with and without TGFβ1. Data are mean ± sem for n = 3 independent experiments; *$p<0.05$, **$p<0.01$, ***$p<0.001$.

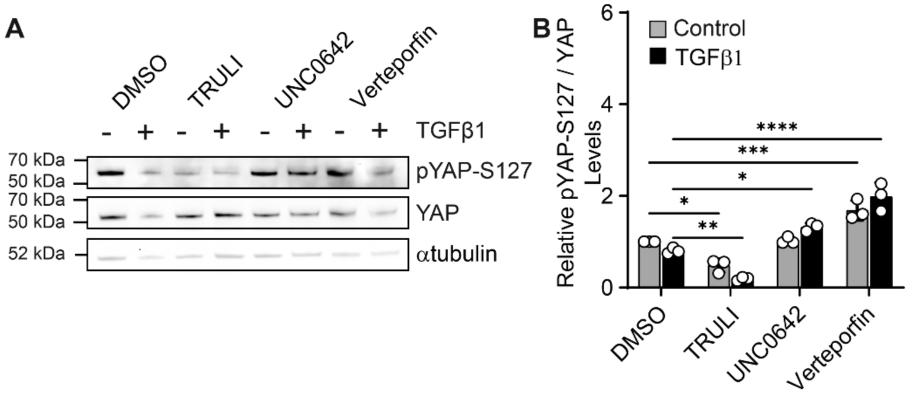

Figure S8. Phosphorylation of YAP is impacted by LATS kinase, G9a, and YAP inhibitors. (A) Western blot for pYAP-S127 and total YAP in NMuMG cells cultured on tissue culture plastic and treated with TGFβ1 and DMSO, TRULI, UNC0642, or Verteporfin. To visualize pYAP-S127 in western blots, 42.9 µg of whole cell lysate was loaded to each well. The high loading resulted in high signal and high contrast images for the αtubulin blot. (B) Densitometric analysis of western blot shown in panel A. Data represent mean ± sem for n = 3 independent experiments; *p<0.05, **p<0.01, ***p<0.001, ****p<0.0001.
